# Islet ultrastructure: past achievements and future directions

**DOI:** 10.1007/s00125-026-06782-7

**Published:** 2026-07-09

**Authors:** Andreas Müller, Solange Aurrecoechea Duran, Patrícia I. Santos, Aubrey Weigel, Isabel Espinosa-Medina, Michele Solimena

**Affiliations:** 1 Molecular Diabetology, University Hospital and Faculty of Medicine Carl Gustav Carus, TU Dresden, Dresden, Germany; 2 Paul Langerhans Institute Dresden (PLID) of Helmholtz Munich at the University Hospital Carl Gustav Carus and Faculty of Medicine, TU Dresden, Dresden, Germany; 3 German Center for Diabetes Research (DZD e.V.), Neuherberg, Germany; 4 Janelia Research Campus, Howard Hughes Medical Institute, Ashburn, VA USA

**Keywords:** Electron microscopy, Islets of Langerhans, Review, Ultrastructure

## Abstract

Islets of Langerhans constitute the endocrine component of the pancreas and comprise the main cell types responsible for the regulation of glucose homeostasis. Beta cells produce and secrete insulin, the principal hormone capable of lowering blood glucose. However, the interplay of beta cells with other endocrine cell types, such as alpha cells and delta cells, is critical for proper blood glucose regulation. The failure of beta cells to produce sufficient amounts of insulin leads to diabetes, a major metabolic disease. Since their discovery by Paul Langerhans using a simple light microscope in 1869, islets have been imaged using many different modalities. From the middle of the 20th century, electron microscopy has been a key tool in resolving the ultrastructure of islet cells, thereby making important contributions to the understanding of protein trafficking in general, and islet cell function as well as dysfunction in diabetes. Recent advances in microscopy techniques as well as the broader availability of analysis tools have elevated islet imaging from two dimensions into three dimensions and enable nanoscale imaging within the tissue context. Here, we review work on islet ultrastructure, from landmark studies published in the 1960s and 1970s to recent advances in imaging. Finally, we discuss open questions and propose ideas for integrating different modalities into structural imaging with the goal of better understanding islet function and dysfunction.

**Graphical Abstract:**

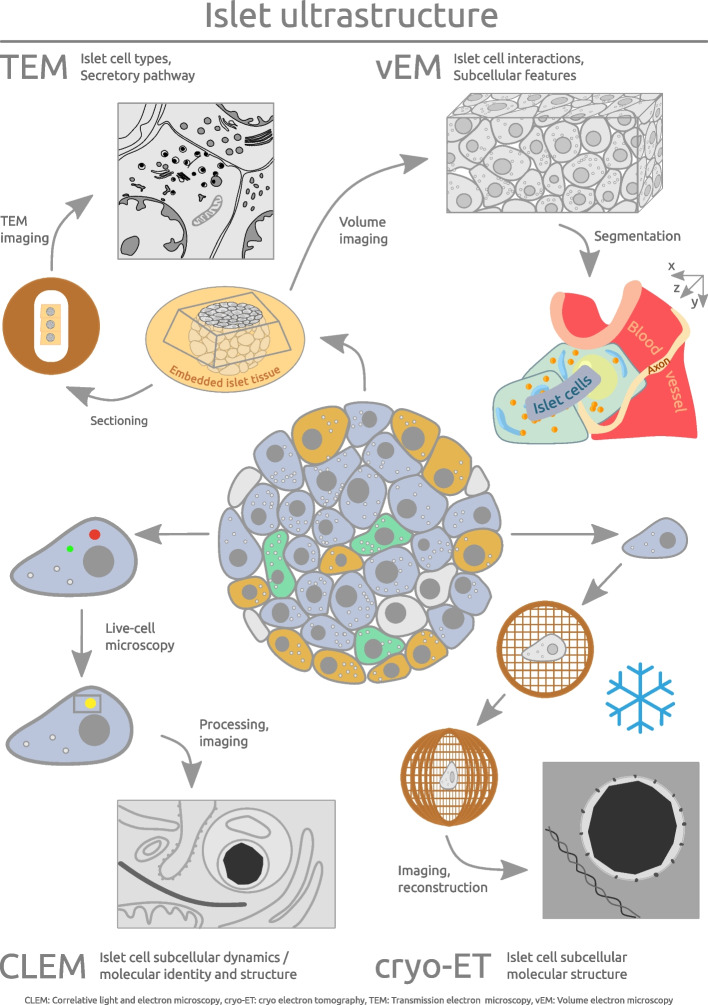

**Supplementary Information:**

The online version contains a slideset of the figures for download available at 10.1007/s00125-026-06782-7.

## Introduction

The islets of Langerhans are micro-organs that were first described by Paul Langerhans in 1869 using light microscopy (LM) [1]. They appear as clusters of cells embedded in the surrounding exocrine pancreas. The islets are complex structures consisting of different peptide hormone-secreting endocrine cell types: alpha cells, which secrete glucagon; beta cells, which secrete insulin; delta cells, which secrete somatostatin; PP cells, which secrete pancreatic polypeptide; and epsilon cells, which secrete ghrelin. Islets are highly vascularised and innervated by the autonomic nervous system. Other cell types present in islets, albeit at lower frequencies than endocrine and endothelial cells, include macrophages, pericytes, stellate cells and fibroblasts. All of these different cell types play pivotal roles in islet function, and the coordinated interactions among endocrine, vascular, neural and immune components enable the precise regulation of blood glucose levels [2, 3].

Shortly after the invention of the transmission electron microscope by Knoll and Ruska in the 1930s [4], the pancreas became a focus of scientists aiming to develop sample preparation techniques to harness the massive increase in resolution that transmission electron microscopy (TEM) provided. This led to the first subcellular images of acinar cells by George Palade [5]. Around that time, the first TEM images of islet cells were also published [6]. Since then, islet structures from multiple species have been imaged using various modalities, including scanning electron microscopy (SEM) and various volume electron microscopy (vEM) techniques.

Electron microscopy (EM) provides detailed structural information on cells and tissues, resolving subcellular features at the nanometre scale, whereas fluorescence microscopy enables the imaging of labelled molecules in living cells and organisms but is limited by the wavelength of light. Notably, the resolution revolution in LM with the invention of various super-resolution techniques has also pushed fluorescent-based optical imaging into the nanometre range [7–9]. In islet research, significant advances have been made by implementing super-resolution techniques to resolve single insulin secretory granules (SGs) [10], reconstruct beta cell mitochondria networks [11] and follow fluorescently labelled glucagon-like peptide-1 (GLP-1) probes [12]. However, EM provides information on both the structure of interest and its complete cellular context, the so-called reference space.

**Fig. 1:**
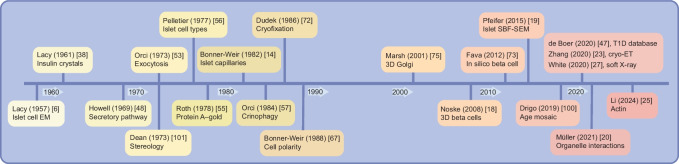
Timeline of major milestones in structural islet imaging. ET, electron tomography; SBF-SEM, serial block-face scanning electron microscopy; T1D, type 1 diabetes. This figure is available as part of a downloadable slideset

The aim of this review is to highlight how structural imaging has contributed to our understanding of islet (cell) function and dysfunction. We provide an overview of methods for ultrastructure visualisation of islets, summarise early 2D EM studies, and describe recent advances in volumetric imaging and analysis, as well as cryogenic EM (cryo-EM) and multimodal approaches, with major milestones highlighted in Fig. 1. Finally, we address current gaps in islet structure imaging and outline potential ideas for future lines of research.

## Technologies used to study islet ultrastructure

**Fig. 2:**
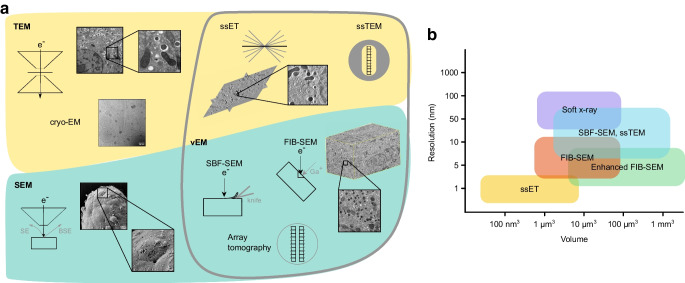
(**a**) Overview of EM techniques for islet imaging. Yellow, TEM-based methods; cyan, SEM-based methods; grey outline, vEM. (**b**) Resolution and volume of different vEM methods. BSE, backscattered electrons; e^−^, electrons; FIB, focused ion beam; SBF-SEM, serial block-face scanning electron microscopy; SE, secondary electrons; ss, serial section. Adapted from [17] under a CC BY 4.0 license (https://creativecommons.org/licenses/by/4.0/). This figure is available as part of a downloadable slideset

The invention of the microscope at the end of the 16th century marked a turning point in life science research. Owing to its ability to magnify and visualise biological structures, it has remained an indispensable tool for biological discovery. The earliest microscopes used visible light to image the sample and were therefore called optical or light microscopes. In the early 20th century, the development of EM introduced the use of electrons instead of photons for imaging, achieving higher resolution. Today, microscopy can broadly be classified into three major categories: LM (optical microscopy); EM; and x-ray microscopy. The distinct physical principles underlying these techniques determine their respective applications and contributions to structural and cellular biology (Table 1). Here, we summarise six decades of high-resolution imaging techniques in islet biology, emphasising methodological advances and open structural questions that guide current and future research. These techniques include TEM, SEM, vEM, cryo-EM and soft x-ray microscopy. An overview of EM-based methods for islet imaging is provided in Fig. 2a.

**Table 1:** Summary of microscopy techniques

Technique	Probe type	Specimen type	Resolution	Imaging depth	Advantages	Limitations	Imaging speed
LM	Photons	Live or fixed specimens	~200 nm (down to a few nanometres with super-resolution)	Surface or shallow volume	Enables live imaging, easy operation	Limited resolution, requires thin samples, usually no cellular background visible	Fast
EM							
TEM/STEM	Electrons	Fixed specimens embedded in resin Thin sections	~1 nm	2D projection or small volume	High resolution, internal ultrastructure visualisation	Requires ultrathin sections (<100 nm) and staining with heavy metals	Slow
SEM	Electrons	Fixed specimens Surface-coated	~1 nm	Surface	High surface detail, 3D-like appearance	Requires staining with heavy metals and gold coating	Slow
vEM	Electrons	Fixed specimens embedded in resin	~4 nm	Volume (3D reconstruction)	High-resolution 3D reconstruction of cellular/tissue structures	Complex data acquisition and processing	Slow
Cryo-EM	Electrons	Frozen-hydrated specimens	~0.2–0.3 nm	Volume	Molecular resolution, preserves native state, no staining needed	Requires cryogenic handling, only small part of cell (below 1%) can be imaged	Slow
x-ray microscopy (TXM/SXM/STXM)	X-rays	Fixed specimens	~15 nm	Surface or volume	Can image thick samples without sectioning, good penetration	Requires specialised facilities and complex set-up	Moderate

STXM, scanning transmission x-ray microscopy; SXM, scanning x-ray microscopy; TXM, transmission x-ray microscopy

### TEM and SEM

Pancreatic islets in situ as well as isolated islets, islet cells and insulinoma cell lines in vitro have mostly been studied by TEM. TEM instruments emit electrons that pass through a system of magnetic lenses and through the sample before reaching a screen and/or camera. The short wavelength of electrons allows for high 2D resolution imaging (down to 1 nm). However, samples need to be very thin and stained with heavy metals in order to be imaged. Combined with autoradiolabelling, this approach enabled the localisation of molecules labelled with isotopes in TEM images and helped elucidate the secretory pathway [13]. Fixation is mostly achieved by immersion in aldehyde-containing solutions; however, these solutions alter ultrastructure. To overcome these limitations, since the 1980s, cryofixation methods have become available and provide much better structural preservation. Especially in the 1960s and 1970s, freeze-fracture TEM was used to visualise cell and organelle surfaces. Later, electron tomography (ET) enabled small 3D volumes to be obtained from thicker TEM sections by gradually tilting the section in the TEM beam, followed by reconstruction of the tilt-series to a tomographic volume. Compared with TEM, in SEM secondary electrons are detected to visualise the surface of cells that have been dried, stained with heavy metals and coated with gold. SEM has been used to image blood vessel networks of islets [14] as well as islet cell primary cilia [15]. In recent years, SEM has also been used to image the surface of the block-face or ultrathin sections using vEM techniques [16].

### vEM

vEM encompasses a family of TEM- and SEM-based methods that enable acquisition of large 3D datasets from cells and tissues [17]. TEM techniques include serial section TEM and serial section ET (ssET). For islet volume reconstruction, ssET has been applied [18]. However, recent developments have shifted the focus toward SEM-based systems. Pancreatic and islet samples have been imaged with serial block-face (SBF)-SEM and focused ion beam (FIB)-SEM systems [19, 20]. In SBF-SEM, a diamond knife within the microscope repeatedly cuts ultrathin sections from the block, followed by imaging of the block-face by SEM, to achieve a 3D volume of the sample. In FIB-SEM systems, the cutting is replaced by ablation of a thin sample layer by an FIB. Depending on the technology used, a voxel size of down to 4 nm and image volumes of several thousand µm^3^ can be imaged [21], which enables imaging of whole islet cells [20] up to complete islets [19, 22]. The range of vEM techniques, together with soft x-ray imaging, provides different combinations of resolution and imaging volume across modalities (Fig. 2b).

### Cryo-EM

Cryo-EM has revolutionised ultrastructure imaging, as it enables the reconstruction of single molecules and molecular complexes at atomic resolution using a combination of advanced sample preparation, imaging and image processing. Cryofixation can be achieved by plunging the sample into liquid ethane or other cryogens (feasible for isolated proteins, organelles or thin cultured cells) or by high-pressure freezing (HPF) (suitable for tissues up to 200 µm thickness, such as isolated islets) [23–25]. The goal of these techniques is the vitrification of water in the tissue and thereby prevention of ice crystal formation, which destroys the ultrastructure of cells. Samples can then be imaged by cryo-TEM or cryogenic ET (cryo-ET) followed by computational processing, such as single particle analysis or subtomogram averaging, to achieve high-resolution structures. Compared with vEM, cryo-EM allows for a much higher resolution down to the molecular level. However, the volumes that can be imaged are usually less than 1% of the volume of a cell.

### Soft x-ray

Not an electron-based technique, soft x-ray imaging [26] enables fast 3D imaging of cell ultrastructure down to 15 nm voxels. Imaging is performed in the water window on frozen single cells with a resolution that enables resolution of SGs, mitochondria and lipid droplets [27].

## Processing techniques and model systems for islet imaging

There are different model systems available to study islet ultrastructure. Traditionally, pancreatic tissue that has been fixed with aldehydes by perfusion or immersion has been used to prepare samples for TEM, SEM or vEM. Notably, pancreas specimens are usually too large to be fixed properly by cryofixation techniques. However, compared with isolated islets, pancreatic tissue preparations preserve innervation and blood vessels, enabling imaging of islet cells in their native tissue context. Islet isolation, in turn, allows for easier metabolic and genomic intervention and for EM fixation by HPF, therefore preventing artefacts introduced by chemical fixation. After HPF, islets are usually processed by freeze substitution, followed by embedding in epoxy resins at room temperature. TEM can be performed after sectioning, or the embedded material can be prepared for vEM. With the invention of protocols for obtaining FIB lamellae of vitrified samples [28], it became possible to perform cryo-EM of isolated islets [29]. However, this is still not a standard method for this material. Cell lines of islet cell types, such as insulinoma or glucagonoma cells [30] and human cell lines [31, 32], are easier to fix by cryofixation methods such as HPF or plunge-freezing, as they usually grow in monolayers. Following cryofixation, the cells can be imaged directly on the EM grid, or lamellae can be cut in order to image regions deeper inside the cells by cryo-TEM or cryo-ET. Notably, insulinoma cell lines are valuable models for gaining insights into islet cell function, although they usually contain fewer SGs and their metabolic regulation is not as tight as in primary tissue. Recent advances in generating pancreas organoids [33] and stem cell-derived islets [34] make these valuable models for islet research in which EM is used to study the structural features of islet development [35] or evaluate the maturation of stem cell islets for islet replacement therapies [36].

## TEM- and SEM-driven discoveries in islets and islet cells

Before the invention of electron microscopes, islets were characterised by LM using various histochemical techniques (reviewed in [37]). These methods enabled the discrimination of beta and alpha cells. With TEM it became possible to image the whole subcellular structure of cells and tissues. TEM has shaped our understanding of the morphology of organelles and even early EM images serve as the gold standard for our understanding of mitochondria morphology or Golgi structure.

**Fig. 3:**
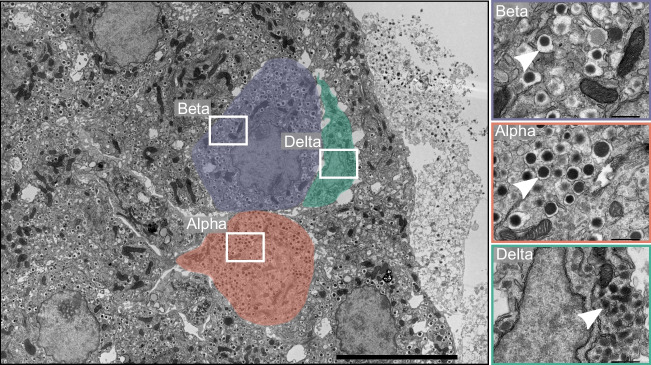
Structural discrimination of mouse islet cell types. TEM image of an isolated mouse islet. The three major islet cell types, beta, alpha and delta cells, are highlighted. Magnified regions show the ultrastructural features of their SGs (arrowheads). Scale bar, 10 µm (main image) or 500 nm (SG insets). This figure is available as part of a downloadable slideset

The first TEM images from pancreatic islets were reported by Paul E. Lacy in the late 1950s [6], from various species. In later publications, he and others presented high-quality TEM images and for the first time described the differences in morphology of insulin SGs from different species including humans [38, 39]. In TEM images of islets, alpha cells and beta cells, which produce glucagon and insulin, respectively, could be distinguished by the appearance of their SGs. Insulin SGs have a crystalline dark core surrounded by a translucent halo, while glucagon SGs have a bigger and darker core without a significant halo (Fig. 3). Depending on the species, insulin SG crystals can have various shapes, ranging from single crystals in mouse and rat beta cells to multiple crystals in human and pig beta cells [38, 40–42], whereas the structure of glucagon SGs does not seem to vary significantly between species. In these early EM works, mitochondria, endoplasmic reticulum (ER) and Golgi were clearly visible, and all membrane systems were resolved. Later on, delta cells, the third major islet cell type, were identified by TEM [43] and shown to produce somatostatin [44]. Compared with alpha cells and beta cells, delta cells are more elongated and neuron-like and their SGs have a more ellipsoid shape. For somatostatin SGs, stronger structural differences between species have been found: in mice, they are easily distinguished from insulin and glucagon SGs by their shape (Fig. 3), whereas in humans somatostatin SGs appear similar to glucagon SGs [45]. Similarly, SGs of PP cells differ between species [46] and discriminating them from somatostatin SGs can be difficult. However, in humans their SGs are much smaller than the SGs of other endocrine cells, enabling the identification of PP cells [47].

**Fig. 4:**
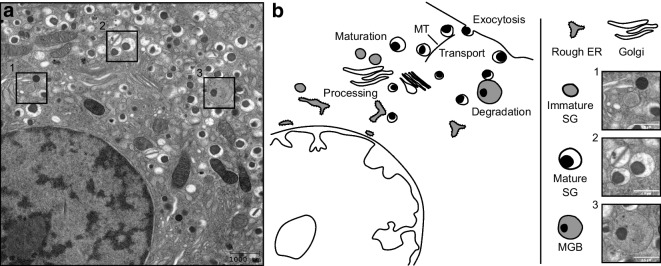
Main stages of the insulin secretory pathway elucidated with major contributions from TEM. (**a**) TEM image of a mouse beta cell. Scale bar, 1000 nm. (**b**) Scheme derived from the TEM image shown in (**a**) depicting the major insulin-processing steps. Notably, SG transport and exocytosis cannot be seen in (**a**) but have been established with TEM. MGB, multigranular body; MT, microtubule. Scale bar, 0.5 µm. This figure is available as part of a downloadable slideset

To investigate the secretory pathway of the islet hormones, autoradiography was introduced to follow the trafficking of proinsulin from the ER through the Golgi apparatus [48, 49]. This technique also enabled the identification of neurotransmitters stored within SGs together with insulin [50]. During its maturation, the structural appearance of the insulin SG changes from a vesicle containing pale and dispersed proinsulin to an electron-dense insulin crystal surrounded by a translucent halo after maturation [51]. Interestingly, knockout of *Slc30a8* (also known as *ZnT-8*) leads to a structurally immature appearance of insulin SGs with normal rates of insulin biosynthesis, content and release [52]. Exocytosis of insulin SGs was first observed by Lacy [6, 38] and then further demonstrated and quantified by Lelio Orci and colleagues in 1973 by freeze-etching EM [53]. Notably, prior to these studies it was debated how peptide hormones were released from the cell and alternative theories claimed that vesicles released their content within the cytosol. Later, TEM together with capacitance measurements enabled the division of the insulin SG population within a cell into functional pools depending on their distance from the plasma membrane [54]. With the introduction of immunogold labelling it became possible to investigate the molecular identity of islet hormones and to identify the other islet cell types [55, 56]. In addition to hormone production and secretion, intracellular degradation was first characterised by EM and immunogold labelling. A main pathway for insulin and also glucagon SG degradation is crinophagy, resulting from the direct fusion of SGs with lysosomes [57, 58]. In beta cells, the resulting crinophagic bodies appear as larger circular structures containing several insulin cores and are therefore also called multigranular bodies. Notably, the insulin crystal shows some resistance to lysosomal enzymes and persists for some time [59], whereas the soluble C-peptide cannot be detected in crinophagic bodies due to its very rapid hydrolysis [57]. Autophagy also plays a role in insulin degradation and was later assessed in samples with optimised structural preservation due to HPF fixation [60]. The role of the cytoskeleton in insulin trafficking and secretion was also suggested early on using EM [61]. Orci and others further revealed the role of microtubules for SG transport [62, 63], while cortical actin was proposed to exert an inhibitory role [62]. Although the transport of insulin SGs by motor proteins along microtubules has been demonstrated in other studies, their role as positive or negative regulators of insulin secretion remains a debated topic [20, 64–66]. In summary, TEM in combination with other techniques has enabled establishment of the main stages of insulin processing (Fig. 4).

In the context of the islet, beta cell polarity has been determined in an elegant study combining serial section histology and TEM, identifying basal and apical poles of beta cells [67]. With immuno-EM, the pancreatic GLUT-2 was localised mainly at the microvilli of beta cells [68], showing the compartmentalisation of glucose sensing into distinct plasma membrane domains. The electrical coupling of islet cells via gap junctions was structurally demonstrated by TEM of ultrathin sections and of freeze-fracture replicas [69, 70]. These data indicated that gap junction abundance changes with glucose stimulation, and changes were later also revealed after beta cell degranulation [70].

The hallmark publications on islet ultrastructure relied on chemical fixation of specimens, a method known to introduce certain artefacts, such as cellular and membrane shrinkage or swelling, as well as extraction of the cytoplasm and the extracellular matrix [71]. Cryofixation techniques, in contrast, enable preservation of cellular ultrastructure. After cryofixation, samples can be processed either for cryo-EM or for room temperature EM by freeze substitution. Early cryofixation by plunge-freezing followed by freeze substitution and plastic embedding already led to satisfactory preservation of islet cells [72], although signs of ice crystal damage can be detected in the presented images. Because islets are relatively large structures, with diameters ranging from 100 μm to 500 µm, plunge-freezing cannot fully prevent ice crystal formation. HPF therefore became the better choice for cryofixation of islets with optimal structural integrity, smooth membranes and non-extracted cytoplasm. Interestingly, in rat islets prepared by HPF, the characteristic halo of insulin SGs was almost completely absent [73], indicating that it is a partial artefact created by chemical fixation and dehydration during embedding. However, depending on the species, this halo is still visible [19].

Compared with TEM, studies with SEM imaging of islets are relatively few. Imaging by SEM provides pseudo-3D information but does not allow for full 3D reconstruction of the imaged structures. By SEM, the islet blood vessel network was shown to be much more complex than that of the exocrine tissue [14]. More recently, human islet cilia were analysed by SEM [15] and part of their molecular composition was assessed by immuno-SEM [74].

## From 2D to 3D

**Fig. 5:**
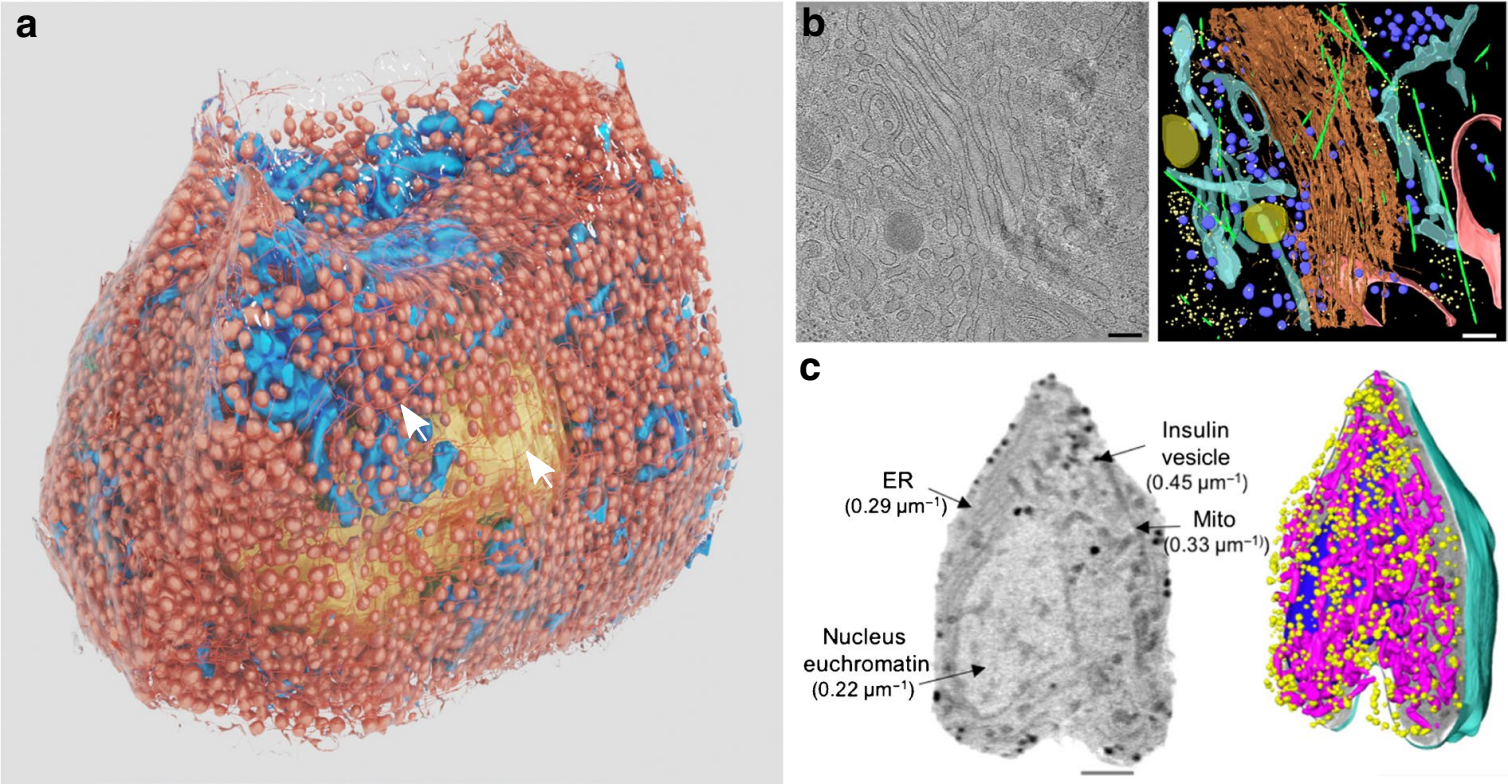
Examples of 3D structural islet imaging. (**a**) 3D rendering of a primary mouse beta cell from enhanced FIB-SEM data. Insulin SGs are shown in orange, mitochondria in blue, nucleus in yellow, and microtubules in red (indicated with white arrows). (**b**) Cryo-ET of INS-1E cell with the corresponding segmentation of organelles. Ribosomes are shown in yellow, ER in brown, microtubules in green, mitochondria in red, Golgi in cyan, transport vesicles in blue, and lysosomes in dark green. Scale bar, 200 nm. Reproduced from [23] under a CC BY 4.0 license (https://creativecommons.org/licenses/by/4.0/). (**c**) Soft x-ray imaging of INS-1E cell with LAC values of different structures and the corresponding segmentation of insulin SGs (yellow), mitochondria (magenta), nucleus (dark blue) and plasma membrane (cyan). Scale bar, 2 μm. Modified from [27] under a CC BY 4.0 license (https://creativecommons.org/licenses/by/4.0/). Mito, mitochondrion. This figure is available as part of a downloadable slideset

TEM provides only 2D information. Even though methods exist to extrapolate quantitative volumetric data out of 2D images (see Analysis tools and open access, below), TEM cannot provide detailed 3D information on cell–cell interactions and subcellular features such as SG distribution, mitochondria morphology or Golgi organisation. However, with ET it became possible to obtain isotropic 3D information out of thin (usually 300 nm) TEM sections, and relatively large 3D volumes of cells can be obtained by reconstructing a series of tomograms using ssET. Due to the high demands related to sample preparation, acquisition and data analysis, ssET requires a lot of skill and time; however, it has led to numerous novel insights into islet cell structure. Using this method, the complete Golgi apparatus of an insulinoma cell line was reconstructed, revealing the organisation of the Golgi stacks, associated vesicles and cytoskeletal elements [75]. In a later work the same authors showed direct connections between Golgi stacks of primary beta cells on glucose stimulation [76], indicating major structural adaptations to meet secretory demands. These studies, to our knowledge, remain the only ones to resolve the Golgi apparatus of a beta cell in 3D at high resolution and have significantly contributed to our knowledge of Golgi structure in general. In another landmark study, Noske et al used ssET to reconstruct complete mouse beta cells and manually segment most of their organelles to analyse SG distribution and mitochondria morphology [18]. This approach also allowed for a detailed analysis of organelle fractions of beta cells and was later accompanied by additional data comparing glucose-stimulated with non-stimulated cells and partial segmentations of beta cell microtubule networks [77]. The development of SEM-based SBF imaging techniques allowed the imaging of much larger volumes compared with ssET. Complete mouse islets were imaged by SBF-SEM [19] followed by multi-scale investigations of the overall islet architecture and volumetric features of alpha and beta cells as well as their granules. With the same techniques, insulin SG maturation times could be determined out of large volumes by determining insulin SG maturation states from the extent of the translucent halo [78]. Additionally, SBF-SEM was used to define the polarity of beta cells in detail, discriminating between basal, apical and lateral surfaces [79]. The authors defined three poles of beta cells: lateral, vascular and avascular poles. They also found that the beta cell primary cilium is mostly located at the avascular pole, indicating its role in paracrine signalling rather than glucose sensing. Notably, SBF-SEM allows for imaging larger fields of view but the z resolution is limited by the physical thickness of the sections, which is ≅25 nm. With FIB-SEM, volumetric data with isotropic voxels can be imaged, although the original technology was limited to small volumes comprising a maximum of one or two cells. The invention of enhanced FIB-SEM [21] overcame these limitations and larger samples could be imaged at high isotropic resolution, albeit at lower speed. With this technique and using tools for organelle-specific segmentation, a reconstruction of microtubule–organelle interactions of complete beta cells in isolated islets was achieved [20] (Fig. 5a). This revealed that beta cell microtubules are non-centrosomal and mostly not Golgi-connected. They are enriched at the cell periphery and interact with insulin SGs. In addition, the interaction of microtubules with mitochondria and/or ER implies that microtubules might coordinate positioning of energy/metabolic hubs with secretion sites. Furthermore, glucose stimulation induces a remodelling of the network, resulting in many more (but much shorter) microtubules, with its overall length remaining stable. Thus, this study demonstrates that microtubule architecture can influence not just trafficking but also local metabolic support for exocytosis, and that a direct influence of metabolism could affect the cellular (ultra)structure. With the rise of these new vEM techniques, such as enhanced FIB-SEM [21, 80], it also became possible to image larger fields of view containing several cells or almost complete islets in their native context, including vasculature and innervation [22]. With these datasets it was possible to reconstruct beta cell primary cilia and investigate their interactions within the islet [81]. The preservation of the whole tissue context led to the discovery of beta and alpha cell cilia axon connections, novel compartments for integrating signals from the peripheral nervous system. Furthermore, these datasets have contributed to a study investigating the targeted release of somatostatin from delta cells towards beta cell cilia [82] and to the exploration of contact sites between insulin SGs and the ER [80, 83]. In addition, with conventional FIB-SEM a striking remodelling of beta cell organelle architecture under different perturbations of ER homeostasis could be observed [84]. This is especially interesting for understanding the pathogenesis of type 2 diabetes since alterations in ER transcriptome and proteome have been observed in individuals with the disease [85].

Some of the studies mentioned previously involve cryofixation for optimal structural preservation, followed by plastic embedding and imaging at room temperature [18–20]. In contrast, with cryo-EM the sample is not embedded in resin but is imaged at cryogenic temperatures in a hydrated state. Most cryo-EM so far has been done in insulinoma cells since they are easier to freeze without inducing ice crystal damage compared with isolated islets or tissue biopsies. The first cryo-EM of INS-1E cells appears in [86], and in a later study this cell line was used to image different regions near the cell cortex and nucleus by cryo-ET to gain insights into the surroundings of insulin SGs (Fig. 5b) [23]. Cryo-EM enables the visualisation of protein complexes in their native environment and is especially suited for imaging cytoskeletal elements. By cryo-ET of insulinoma cells and mouse islets, the remodelling of actin filaments upon glucose stimulation could be shown at the single-filament level [25]. Cryo-EM studies of islets are still quite rare. However, a recent study demonstrates high-resolution cryo-ET of selected regions of mouse islets [29]. Compared with SBF-SEM and FIB-SEM, the volumes that can be imaged with cryo-ET are very small, with a thickness of 100–300 nm, which is the diameter of an SG. However, serial lift-out techniques enable high-resolution imaging of a series of tomograms from a single small organism [87] and could be adapted to imaging islets.

Volumetric structural imaging of cells is also possible with x-rays. Soft-x-rays have been used to investigate subcellular features of insulinoma and glucagonoma cell lines [27, 88, 89]. Although the method is limited in resolution to approximately 50 nm, it allows for fast imaging of single cells and the resolution of insulin SGs, mitochondria and the nucleus (Fig. 5c). Furthermore, it is possible to obtain a direct readout on SG maturation with the linear absorption coefficient (LAC) values, which allow for the investigation of SG subpopulations under different stimuli [90].

## Multimodal approaches

**Fig. 6:**
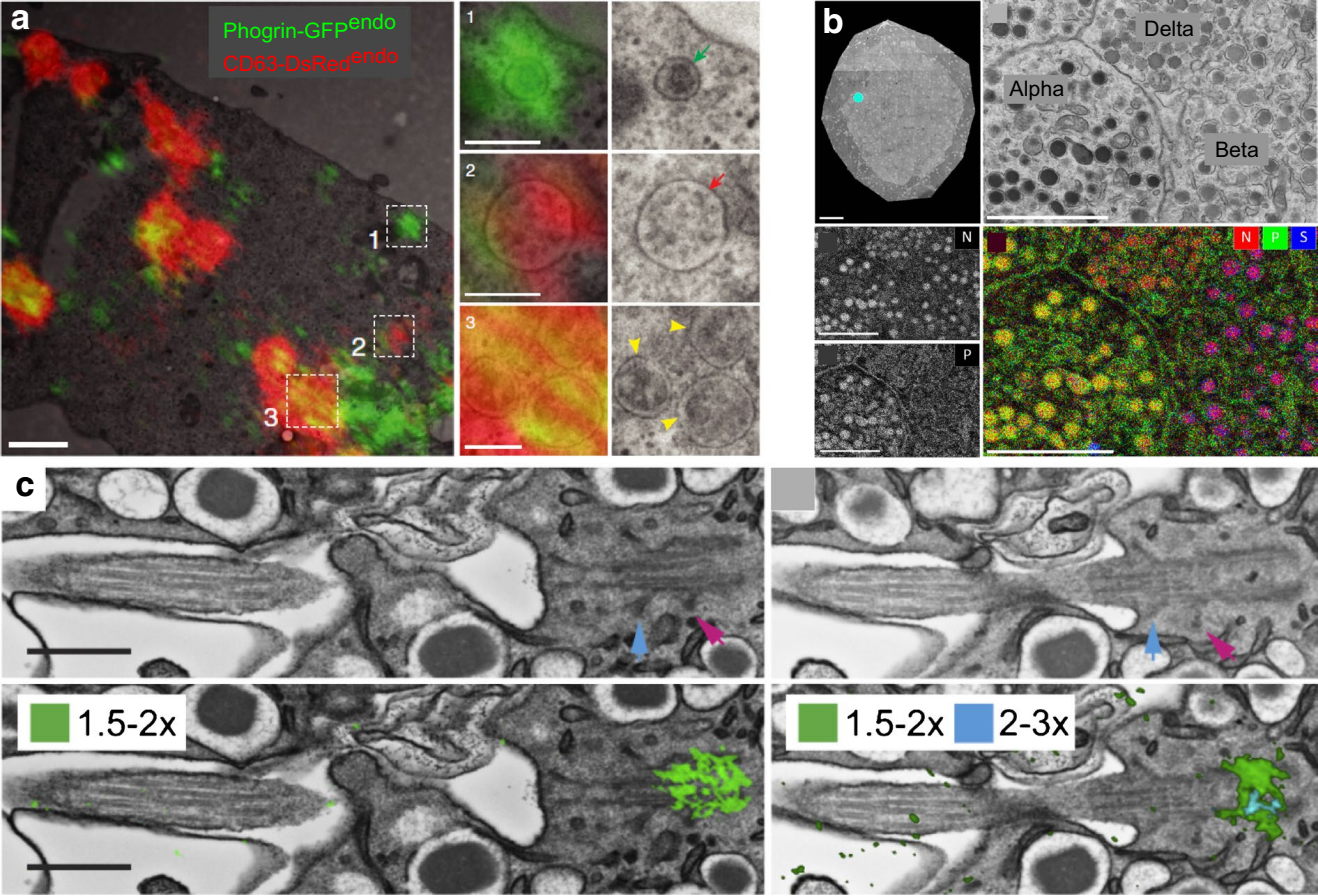
Examples of correlative and multimodal islet imaging. (**a**) CLEM of INS-1E cell with insulin SG labelled by Phogrin-GFP (green) and lysosomes by CD63-DsRed (red). The magnified regions show different stages of SG degradation. Arrowheads indicate insulin SG (green), lysosome (red) and lysosome containing insulin (yellow). Scale bar, 1 µm (main image) or 300 nm (insets). Modified from [95] under a CC BY 4.0 license (https://creativecommons.org/licenses/by/4.0/). (**b**) ColorEM of rat pancreas tissue. The overview STEM image shows a whole rat pancreatic islet, with the region of interest indicated by a blue dot; magnification of this region shows an alpha, beta and delta cell. The corresponding N and P images show the discrimination of cell types by elemental mapping, and the NPS image is a composite image including three elements (nitrogen, phosphorus, sulfur). Scale bar, 50 µm (overview) or 2 µm (other images). Modified from [143] under a CC BY 4.0 license (https://creativecommons.org/licenses/by/4.0/). (**c**) SEM (top row) and correlated SEM with multi-isotope imaging mass spectroscopy (bottom row) of mouse pancreas tissue. The isotope signal is present at the basal body of a beta cell primary cilium that is followed through two adjacent sections. ^15^N/^14^N thresholds: 1.5–2× (green) and 2–3× (cyan) the natural ^15^N/^14^N of 37 × 10^4^. Arrows indicate transition fibres (blue) and basal root (pink). Scale bar, 300 nm. Reproduced from [100] with permission from Elsevier. This figure is available as part of a downloadable slideset

Although EM provides structural information and the complete cellular background, it lacks information such as molecular and dynamic signatures. Multimodal and correlative approaches overcome these limitations by combining EM with other microscopy techniques or even methods that are not directly related to imaging. Early techniques such as radiolabelling and immunogold enabled the direct localisation of labelled proteins in EM images. Additionally, using consecutive sections for histological staining and EM can provide a correlative approach [38]. With correlative light and EM (CLEM), structures labelled by fluorescent dyes or proteins are correlated to their EM counterparts. This can be achieved by different workflows [91]. With CLEM, islet cell types can be characterised by both their ultrastructure and the molecular identity of their cargoes [92]. This has also enabled a precise calculation of insulin SG half-life by combining age-defined SG labelling [93] with TEM [94]. CLEM also contributed to an in-depth analysis of insulin SG degradation [95] (Fig. 6a). Recently, CLEM has contributed to unravelling the trafficking of GLP-1 receptors towards endosome–ER–mitochondria contact sites [96], elucidating the mechanism by which GLP-1 restores beta cell function. Other than fluorescence microscopy, EM can be combined with elemental or isotope mapping to give information on the elemental composition of structures or the incorporation of labelled amino acids. So-called colorEM [97] allows for distinction of islet cell types by their elemental profile (Fig. 6b) and is especially interesting for the identification of polyhormonal cells [98]. A similar approach has been used to characterise the content of Zn^2+^ and Ca^2+^ in insulin SGs in cells and isolated SGs [99]. Using SEM correlated with multi-isotope imaging mass spectroscopy, it was possible to determine the protein turnover at the cell and organelle level, revealing that most islet cells become postmitotic while only a small fraction is capable of proliferation [100]. Furthermore, proteins of certain organelles, such as primary cilia, have distinct ages, indicating specific roles of older protein assemblies in islet cell function [100] (Fig. 6c).

## Analysis tools and open access

Islet morphology analyses in 2D and 3D images can be performed applying ‘classical’ stereological methods [54, 101, 102]. Morphometric grids are placed on top of electron micrographs to estimate volume, surface area, number of cells and organelles such as insulin SGs. Although stereological analyses provided a simple analysis method they lacked certainty on the correct sectioning in 2D and the 3D context of the islet. In later studies, analysis of insulin SG abundance and distributions in TEM images was performed by automated segmentation and in silico modelling, enabling a comprehensive quantitative assessment of insulin SG number and distribution in rat beta cells [73].

Advances in vEM have substantially increased the size of raw images and thus driven the need for new computational strategies for image processing and analysis since tasks such as manual annotation of all SGs in a whole-cell volume are exceedingly time-consuming. The development and applicability of methods based on artificial intelligence (AI) has also fostered automation of cell segmentation in islet research. For the reconstruction of microtubule–organelle interactions of beta cells [20], a plethora of methods from manual to deep-learning-based segmentation was used to segment and analyse seven different organelles [103]. For the segmentation of insulin SGs (and potentially other endocrine SG types) shape-aware methods such as StarDist [104] show excellent results because they take into account the spherical nature of SGs. Since the segmentation of insulin SGs in 2D and 3D EM data has been of special interest in recent years, several other automated approaches have been developed [105, 106]. For other organelles, such as mitochondria, models that have been pre-trained on a large amount of data have been made available in ready-to-use tools such as ‘empanada’ [107], a plugin for Napari [108], and perform well in segmenting islet cell mitochondria. Additionally, segmentation of islet cell organelles can be facilitated by the combination with elemental mapping [109].

With the rise of cryo-EM and vEM, platforms enabling the reuse of datasets under open access license have been established and also contain data for islet cells. The most well-known repositories are currently EMPIAR [110] and OpenOrganelle [80]. They assist researchers in finding both raw datasets and labels for training segmentation algorithms and contain EM data for islets and islet cells. Nanotomy.org provides TEM open access data for pancreas specimens from people with type 1 diabetes. Additionally, a selection of 2D TEM images of islet cells is hosted by Cell Image Library [111]. Information on these platforms is summarised in Table 2.

**Table 2:** Open access platforms with EM pancreatic datasets

Platform	Dataset	Link
EMPIAR	FAST-EM from rat pancreatic tissue	https://www.ebi.ac.uk/empiar/EMPIAR-12193/
	FAST-EM from rat pancreatic tissue	https://www.ebi.ac.uk/empiar/EMPIAR-12174/
	Cryo/RT from mouse islets	https://www.ebi.ac.uk/empiar/EMPIAR-11469/
OpenOrganelle	Enhanced FIB-SEM from mouse pancreas and islets	https://openorganelle.janelia.org/datasets/jrc_mus-pancreas-1, https://openorganelle.janelia.org/datasets/jrc_mus-pancreas-2, https://openorganelle.janelia.org/datasets/jrc_mus-pancreas-3, https://openorganelle.janelia.org/datasets/jrc_mus-pancreas-4
Nanotomy	Variety of large-scale 2D EM islet datasets	https://nanotomy.org/OA/index.html
BetaSeg	Segmentation masks for mouse beta cell organelles from [20]	https://betaseg.github.io/

FAST, fast automated scanning TEM; RT, room temperature

## Islet structure in diabetes

Changes in the abundance of the different islet cell types and changes of islet architecture in type 1 or type 2 diabetes have mostly been assessed by LM methods [112]. Depending on the disease and its progression, the destruction or degranulation of beta cells, infiltration of islet tissue with immune cells and overall changes in islet organisation can be observed. The majority of the work on ultrastructural changes of islet cells in diabetes has been done in animal models [113–117]. In a mouse model of type 2 diabetes in which transgenic mice express a human neonatal diabetes mutation (Kir6.2-V59 M), reduced numbers of insulin SGs were observed in addition to large vacuolar structures in the beta cell cytosol with no noticeable changes in the ultrastructure of the other endocrine cells [118]. The ultrastructure of insulin SGs in *db*/*db* mice, a model of type 2 diabetes, was investigated by scanning TEM (STEM) to collect 3D information [119]. In addition to a decrease in the number and electron density of insulin SGs being observed, these granules were found to be closer to the plasma membrane compared with control beta cells. While most publications have focused on ultrastructural changes of beta cells in type 2 diabetes, changes in delta cell volume and extension of their filopodia have also been observed with TEM [120] in a diabetic mouse model. In NOD mice, a type 1 diabetes model, crinophagic bodies were identified as being connected to diabetes progression evaluated by TEM [121]. Furthermore, EM in many studies mostly accompanies results obtained with other approaches. However, there also exist publications that have included material from individuals with type 2 diabetes [122–125]. Data from those studies provide structural evidence for alterations in mitochondria and ER, as well as amyloid deposition upon type 2 diabetes. The largest EM database to date is related to type 1 diabetes [47] and contains large-scale 2D images of complete islets in pancreas specimens from the nPOD biorepository [126]. Major findings in these datasets are the increased presence of endocrine cells containing zymogen granules and the increased number of immune cells in specimens from people with type 1 diabetes. Notably, such a comprehensive database is currently not available for specimens from donors with type 2 diabetes and would be highly valuable for investigating structural changes in type 2 diabetes.

## Conclusions and outlook

Since the invention of electron microscopes, EM has provided significant insights into islet structure and function (Fig. 5). However, many aspects of islet structure remain unexplored. Importantly, most previously mentioned publications focus on imaging of beta cells. Although insulin production and secretion by these cells are the major factors for glucose homeostasis, the other islet cell types also play essential roles. Beta cells have been fully reconstructed in 3D but comparable information for other islet cell types is lacking and would provide valuable insights into their physiology. While full 3D reconstructions of these other islet cell types have not yet been achieved, small volumes of delta cells have been imaged by ET and FIB-SEM to investigate delta cell paracrine signalling [127] and interactions with beta cell cilia [82]. Comprehensive analyses of islet and non-endocrine tissue are also lacking, including a complete map of the interactions between blood vessels, nerves and islet cells.

In terms of islet structure in diabetes, the only comprehensive EM database currently available is the aforementioned nanotomy database for type 1 diabetes specimens hosted by the Giepmans lab [47]. Establishing a comparable dataset for type 2 diabetes, particularly using material or samples collected throughout disease progression, such as in the LIving DOnor PAncreas COhort (LIDOPACO) [128], would be extremely valuable. Importantly, future ultrastructural studies of type 2 diabetes should be designed to capture defined disease stages, distinguishing insulin resistance from compensatory beta cell adaptation and failure with organelle dysfunction and loss of secretory capacity. Recently, publications showing alterations in the non-endocrine part of the pancreas, such as the exocrine tissue and immune cells [129], islet pericytes and capillaries [130, 131], and islet innervation [132, 133] highlight the importance of studying these compartments to gain a better understanding of diabetes. Additionally, in a recent study, explainable AI has identified changes in fluorescent images of specimens from people with and without type 2 diabetes that were not connected to beta cells [128], making a further case for also investigating the structure of non-endocrine compartments in diabetes.

The structural changes in islet cells during development appear to be massively understudied, with only a few publications investigating pancreatic development by TEM [134–136]. One of these studies showed maturation of cell–cell junctions during the process of pancreatic branching morphogenesis via TEM but endocrine cell changes were not assessed [134]. Other studies established the presence of different endocrine cell types in developing human islets based on the structure of different SGs [135, 136]. By combining immunogold labelling of specific peptides and TEM it was demonstrated that many differentiating endocrine cells are multihormonal and progressively become monohormonal as the pancreas matures [135]. While these studies establish fundamental features of fetal endocrine cells, they lack the 3D context necessary to define the changes in endocrine cell distribution and cell–cell contacts as the islets mature. This is important, as previous studies have found that spatiotemporally regulated cell sorting, islet morphology and endocrine differentiation are directly related [137]. Thus, gaining volumetric EM resolution (e.g. via FIB-SEM) to study islet development would be beneficial. Given the smaller size of endocrine islets during development, FIB-SEM volumes could comprise larger regions including endocrine and other components fundamental for proper islet maturation, such as the vasculature and innervation [138, 139]. Defining ultrastructural features of immature and mature islet cells in 3D would be valuable for making better stem-cell-derived islets for replacement therapy in type 1 diabetes.

The recent works using cryo-EM to investigate beta cell structure are highly promising but have not yet resulted in high-resolution structures of protein complexes important for islet function. Notably, the reason for the species-dependent differences in insulin SG crystal shapes first documented in the 1950s are still unclear and cryo-EM could be used to obtain high-resolution structures of isolated insulin SGs [24, 140]. With the advances in in situ cryo-EM we are confident that so-called ‘visual proteomics’ of islet cell SGs or other organelles will be available in the near future [141].

Connecting EM data with other multimodal approaches, such as spatial transcriptomics, proteomics or functional islet data, would be extremely valuable for diabetes research. Correlation of spatial transcriptomics with EM data has so far only been demonstrated in brain tissue [142] and is technically challenging. Expanding the application of these strategies to the pancreas would help establish the link between molecular and ultrastructural changes under healthy and disease conditions.

In summary, EM has shaped our understanding of islet structure and function over decades. New possibilities in large-scale 2D and 3D imaging, combined with AI-supported image analysis and combinations with different modalities, will enable us to tackle outstanding questions in islet structure in health and disease in the near future.

## Supplementary Information

Below is the link to the electronic supplementary material.

Slideset of figures (PPTX 7.58 MB)

## Abbreviations

AI: Artificial intelligence
CLEM: Correlative light and electron microscopy
Cryo-EM: Cryogenic electron microscopy
Cryo-ET: Cryogenic electron tomography
EM: Electron microscopy
ER: Endoplasmic reticulum
ET: Electron tomography
FIB: Focused ion beam
GLP-1: Glucagon-like peptide-1
HPF: High-pressure freezing
LAC: Linear absorption coefficient
LM: Light microscopy
SBF: Serial block-face
SEM: Scanning electron microscopy
SG: Secretory granule
ssET: Serial section electron tomography
STEM: Scanning transmission electron microscopy
TEM: Transmission electron microscopy
vEM: Volume electron microscopy
