# Supplementary material for: Islet ultrastructure: past achievements and future directions
Source: Diabetologia. 2026 Jul 9;69(9):2379–95. doi: 10.1007/s00125-026-06782-7 (PMC13424715; doi:10.1007/s00125-026-06782-7)
Supplement: Supplementary file 1 — Slideset of figures (PPTX 7.58 MB) [file 125_2026_6782_MOESM1_ESM.pptx]

## Slide 1
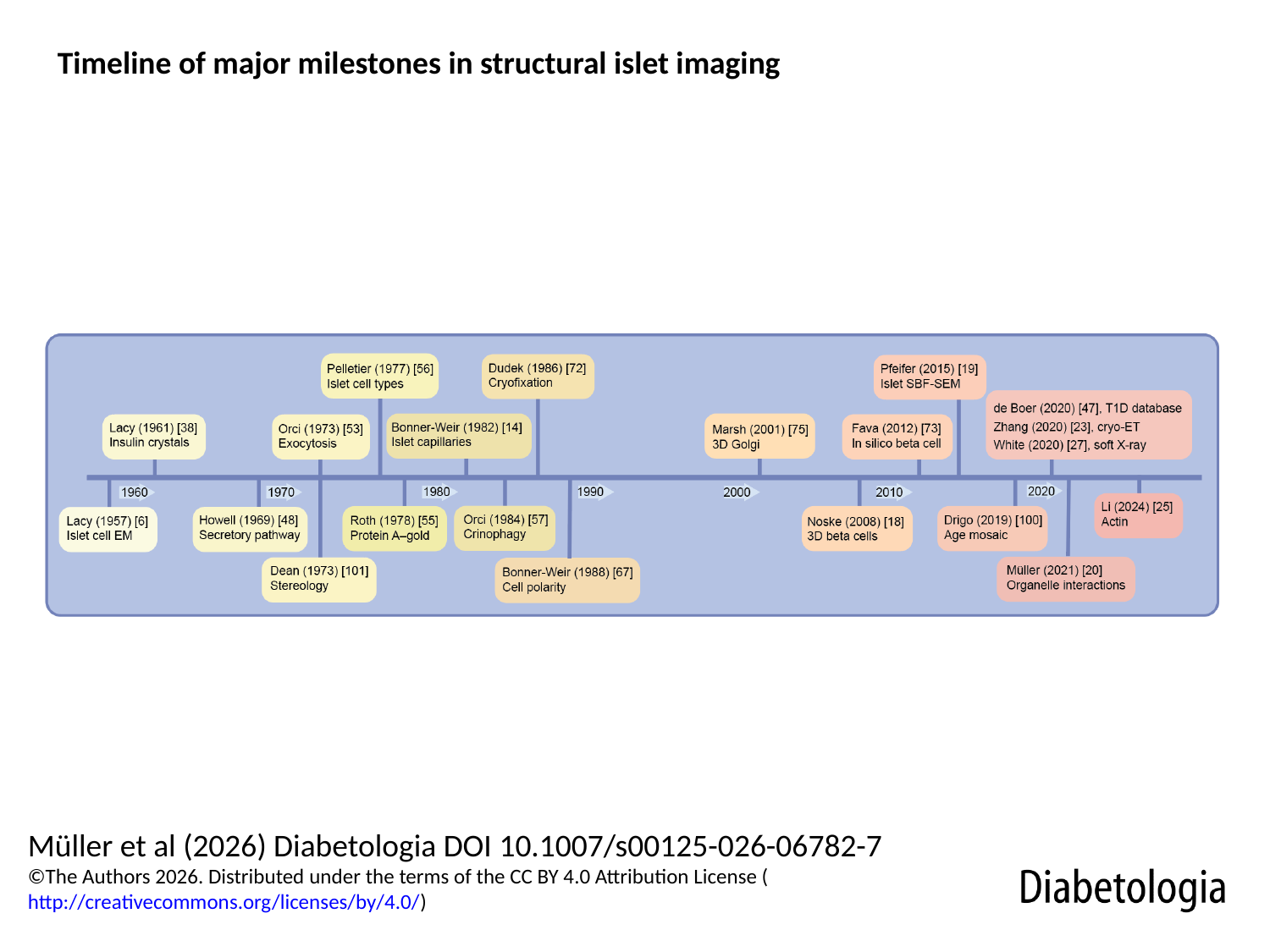

Timeline of major milestones in structural islet imaging
Müller et al (2026) Diabetologia DOI 10.1007/s00125-026-06782-7
©The Authors 2026. Distributed under the terms of the CC BY 4.0 Attribution License (http://creativecommons.org/licenses/by/4.0/)

## Slide 2
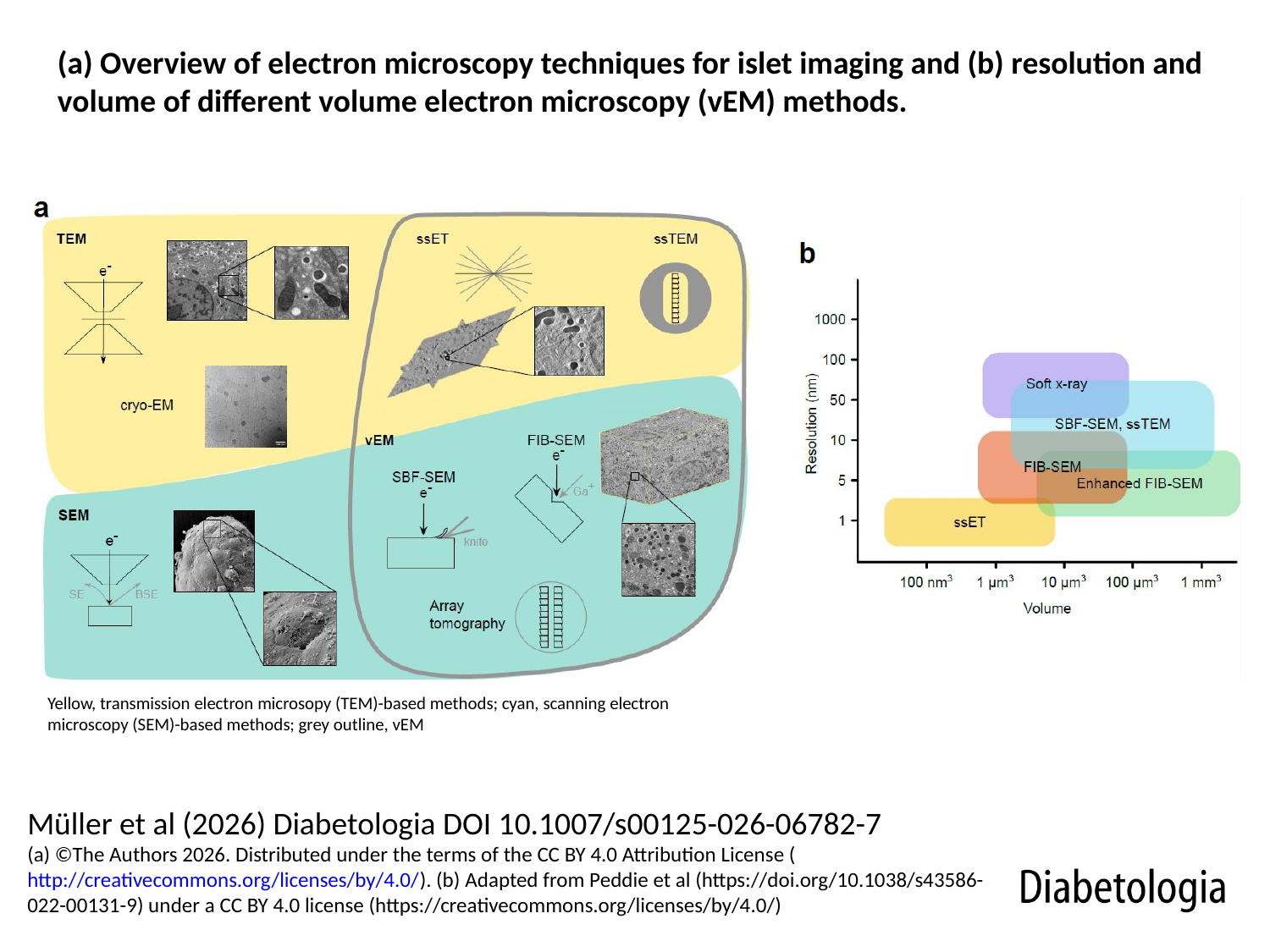

(a) Overview of electron microscopy techniques for islet imaging and (b) resolution and volume of different volume electron microscopy (vEM) methods.
Yellow, transmission electron microsopy (TEM)-based methods; cyan, scanning electron microscopy (SEM)-based methods; grey outline, vEM
Müller et al (2026) Diabetologia DOI 10.1007/s00125-026-06782-7
(a) ©The Authors 2026. Distributed under the terms of the CC BY 4.0 Attribution License (http://creativecommons.org/licenses/by/4.0/). (b) Adapted from Peddie et al (https://doi.org/10.1038/s43586-022-00131-9) under a CC BY 4.0 license (https://creativecommons.org/licenses/by/4.0/)

## Slide 3
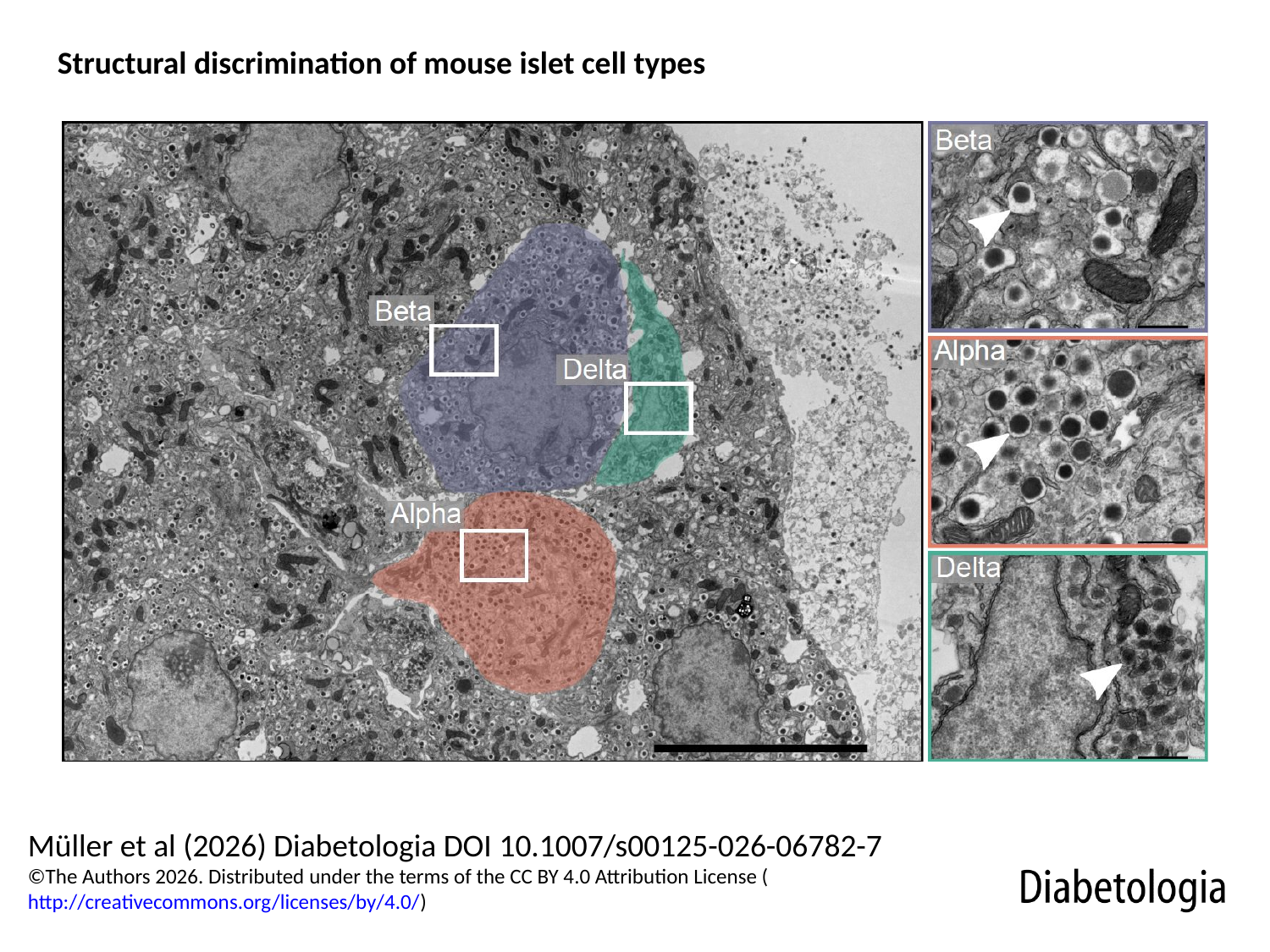

Structural discrimination of mouse islet cell types
Müller et al (2026) Diabetologia DOI 10.1007/s00125-026-06782-7
©The Authors 2026. Distributed under the terms of the CC BY 4.0 Attribution License (http://creativecommons.org/licenses/by/4.0/)

## Slide 4
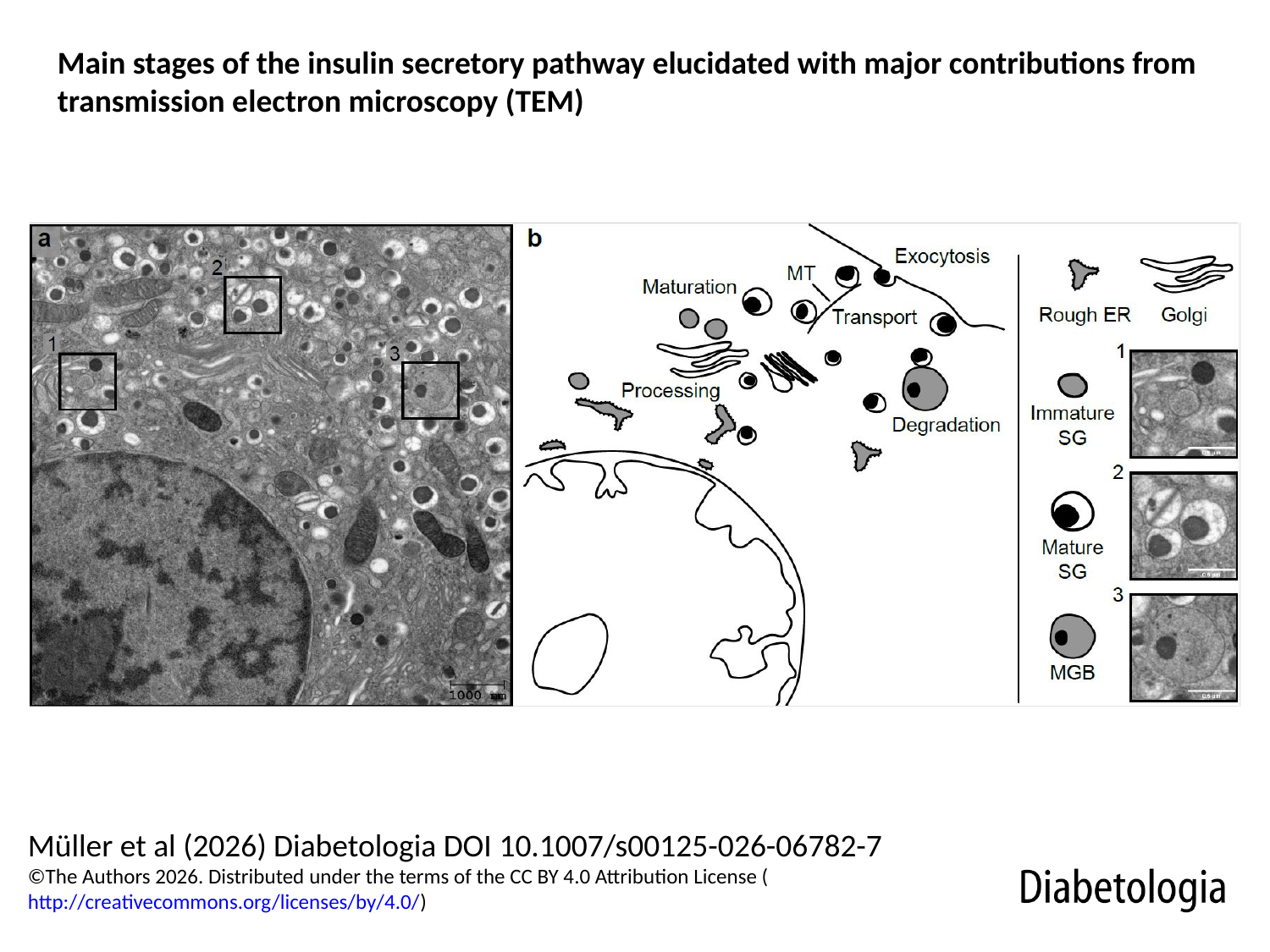

Main stages of the insulin secretory pathway elucidated with major contributions from transmission electron microscopy (TEM)
Müller et al (2026) Diabetologia DOI 10.1007/s00125-026-06782-7
©The Authors 2026. Distributed under the terms of the CC BY 4.0 Attribution License (http://creativecommons.org/licenses/by/4.0/)

## Slide 5
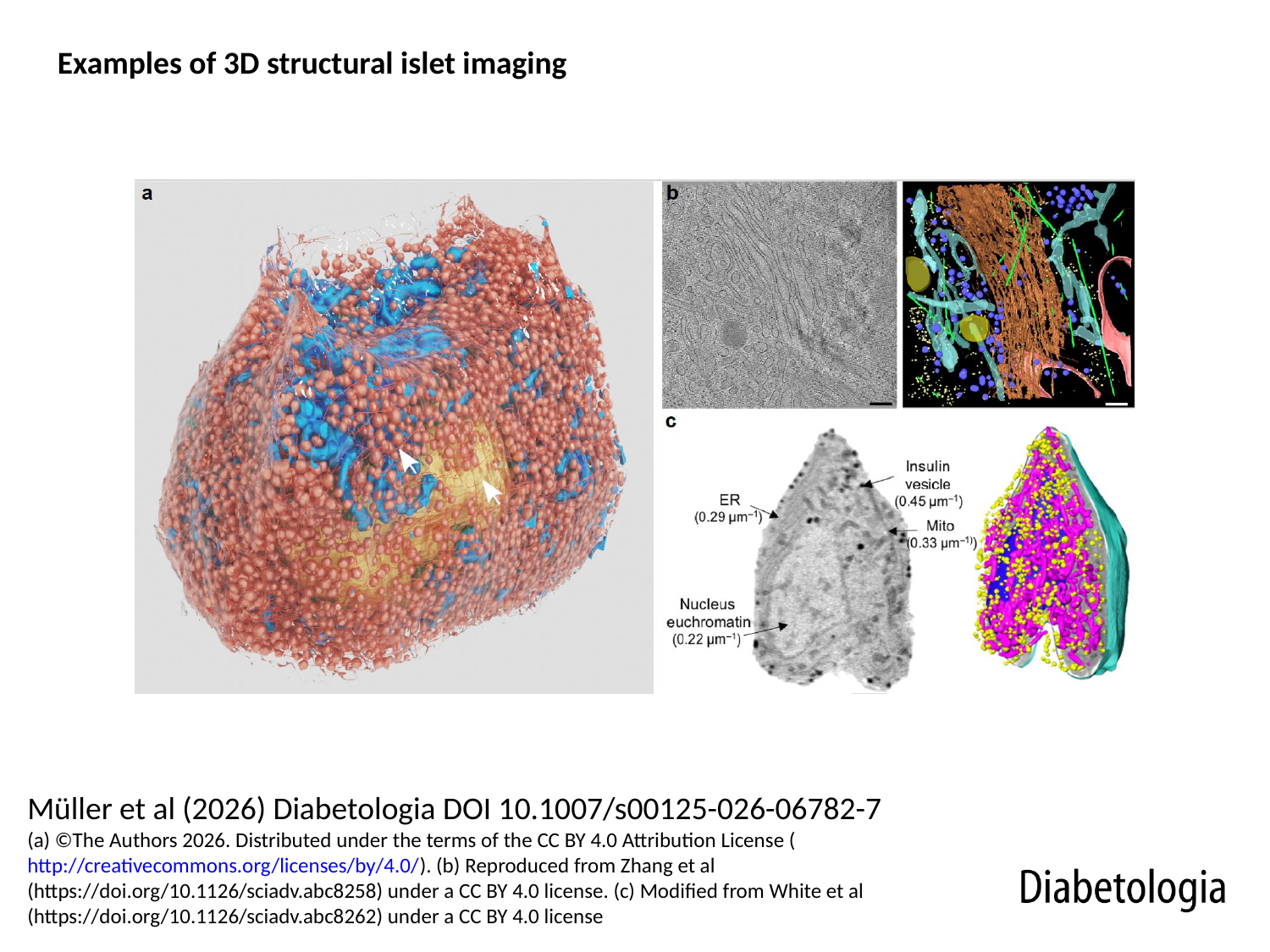

Examples of 3D structural islet imaging
Müller et al (2026) Diabetologia DOI 10.1007/s00125-026-06782-7
(a) ©The Authors 2026. Distributed under the terms of the CC BY 4.0 Attribution License (http://creativecommons.org/licenses/by/4.0/). (b) Reproduced from Zhang et al (https://doi.org/10.1126/sciadv.abc8258) under a CC BY 4.0 license. (c) Modified from White et al (https://doi.org/10.1126/sciadv.abc8262) under a CC BY 4.0 license

## Slide 6
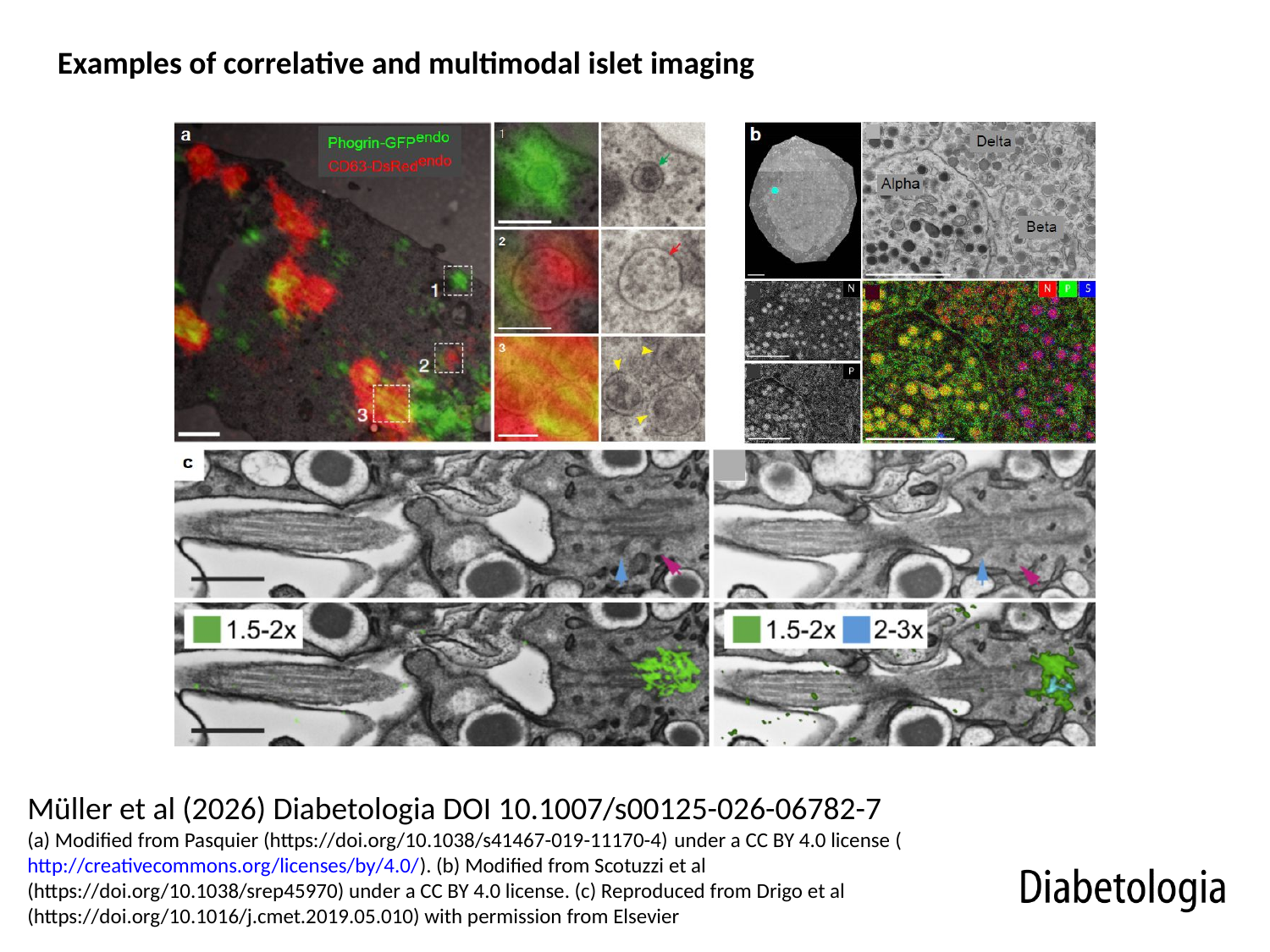

Examples of correlative and multimodal islet imaging
Müller et al (2026) Diabetologia DOI 10.1007/s00125-026-06782-7
(a) Modified from Pasquier (https://doi.org/10.1038/s41467-019-11170-4) under a CC BY 4.0 license (http://creativecommons.org/licenses/by/4.0/). (b) Modified from Scotuzzi et al (https://doi.org/10.1038/srep45970) under a CC BY 4.0 license. (c) Reproduced from Drigo et al (https://doi.org/10.1016/j.cmet.2019.05.010) with permission from Elsevier
